# Carriage of multidrug-resistant organisms in a tertiary university hospital in Albania—a point prevalence survey

**DOI:** 10.1186/s13756-016-0128-1

**Published:** 2016-08-05

**Authors:** Falzon A. Parascandalo, P. Zarb, E. Tartari, D. Lacej, S. Bitincka, O. Manastirliu, D. Nika, M. A. Borg

**Affiliations:** 1 Infection Control Unit, Mater Dei Hospital, Birkirkara Bypass, Msida, 2080 MSD Malta; 2 University Hospital Centre, ‘Mother Teresa’, Tirana, Albania

**Keywords:** Infection-prevention, Antimicrobial-resistance, Meticillin resistant *staphylococcus aureus* (MRSA), Enterobacteriaceae, ESBL

## Abstract

**Background:**

Antimicrobial resistance has been recognised as a serious global Public Health problem. Prevalence of Multiple-Drug-Resistant (MDR) organism carriage in Albania is largely unknown since no national surveillance system is in place and few publications are accessible in the literature.

**Methods:**

A 1-day point-prevalence-survey (PPS) screening for nasal methicillin-resistant Staphylococcus aureus (MRSA) and rectal MDR Gram-negative carriage was carried out at the high-dependency wards in the country’s only tertiary care hospital, in Tirana.

**Results:**

A total of 106 nasal and 104 rectal swabs were collected. 14.2 % of patients (95 % Confidence Interval [95 CI]: 8.1–22.3 %) were MRSA nasal carriers. Resistance to aminoglycosides and fluoroquinolones was common in these isolates (≥80 %) but no resistance was identified against glycopeptides, nitrofurantoin and the relatively newer agents, tigecycline and linezolid. Fifty Enterobacteriaceae isolates were cultivated from 33 of 104 screened patients (31.7 % [95 CI: 22.9–41.6 % 95 CI]). The prevalence of Extended Spectrum Beta-Lactamase (ESBL) production in Enterobacteriaceae was 41.3 % (95 CI: 31.8–51.4 %). The two more commonly isolated Enterobacteriaceae were *E. coli* ([*n* = 28], 24 ESBL positive; 1 AmpC positive and 3 without an identified mechanism of resistance) and *Klebsiella pneumoniae* ([*n* = 13], all ESBL positive; 1 also AmpC and metallo-β-lactamase (MBL) positive). Susceptibility to carbapenems (≥98 %), fosfomycin (90 %) and amikacin (70 + 20 % intermediate) was high but a high level of resistance to all other agents tested was noted. Non-fermenting Gram-negative bacilli were less commonly isolated {22 isolates: *Acinetobacter baumannii* (9); *Pseudomonas aeruginosa* (8) and *Stenotrophomonas maltophilia* (5)}.

**Conclusion:**

Although a significant rate of MRSA carriage was identified, the main resistance challenge in Albania appears to be linked with Gram-negative organisms, particularly ESBL in Enterobacteriaceae.

## Background

Antimicrobial resistance (AMR) is a serious, ever-growing, problem [1, 2]. The World Health Organization (WHO) has declared this as a priority issue, and is developing global action plans to scale up the fight in combating AMR [3].

Carriage and transmission of multidrug-resistant organisms (MDRO) have become a public health concern worldwide [4, 5]. These include, both Gram-positive organisms such as MRSA as well as multidrug-resistant Gram-negative (MRGN) organisms; the spread of Enterobacteriaceae producing extended spectrum beta-lactamase (ESBL) and carbapenemases (CPE) have emerged as a major clinical challenge [6, 7].

Albania is a small country in south-eastern Europe with a population of about 3,500,000 inhabitants. The University Hospital Centre ‘Mother Teresa’ of Tirana (QSUT) is the largest hospital in the country and the only tertiary-care referral centre for acute and critical patients. It incorporates 1555 beds, of which, around 110 are classified as intensive-care. Surveillance systems for Healthcare Associated Infections (HAIs) caused by MDRO have been established in most European countries; the highest proportions of resistant isolates have been reported in the Mediterranean countries [8]. However, the extent to which the healthcare systems in Albania have been affected is largely unknown. The first PPS on HAI conducted in QSUT found a high prevalence of nosocomial infections in intensive care units (31 %) [9].

This paper reports the results of a 1-day PPS to estimate prevalence of carriage of MRSA and MRGN in ‘Mother Teresa’ Hospital Tirana, Albania. The objectives of this survey were to: 1) assess the prevalence of nasal carriage of MRSA in QSUT; 2) assess the prevalence of rectal carriage of MRGN, 3) describe the susceptibility profiles of the isolates; 4) provide guidance on infection prevention and control measures to be undertaken.

## Methods

A delegation of 3 representatives from the Infection Control Unit in Mater Dei Hospital (MDH), Malta, visited QSUT in April 2015 in order to carry out a PPS. Patients with a higher risk of acquiring MDROs were targeted as the sampling cohort and included patients from Central Intensive Care Unit, high dependency wards, haematology, haemodialysis patients from the renal unit and long stay/chronic patients from medical and surgical wards throughout the hospital. Information on date of admission, diagnosis, previous and current antibiotic treatment including the type of antibiotic, was collected.

Verbal consent was obtained from the patients before sampling. Each patient was sampled nasally, and rectally, using Amies Charcoal swabs (Transwab®MWE) pre-moistened with sterile saline. The total number of samples obtained was 106 nasal swabs and 104 rectal swabs; 2 patients accepted nasal, but refused rectal sampling.

The swabs were inoculated onto various chromogenic plates. Initial growth vs. no growth was noted following an overnight incubation, with further work-up of positive specimens. Resistance thresholds determined by the European Committee on Antimicrobial Susceptibility Testing (EUCAST) were used. Identification and sensitivity testing were performed on all the different organisms isolated, using the automated VITEK®2-compact system.

### Quality control

Quality control was performed on the sterile saline, catalase, coagulase and oxidase used, as well as the Agar media. For MRSA the following strains were utilised; ATCC 43300 (MRSA) as a positive control and ATCC 25932—*Staphylococcus aureus* as a negative control. For MRGN the organism strains used were: ATCC BAA1705—*Klebsiella pneumoniae* KPC, ATCC 700603—*Klebsiella pneumonia*e ESBL (SHV-18) and ATCC 27853-*Pseudomonas aeroginosa* as positive controls and ATCC 25922—*Escherichia coli* as a negative control.

### Nasal swabs for MRSA

The presence of MRSA was tested for on any pink colonies grown on a MRSA Select ™II chromogenic Agar by performing a sub-culture onto Blood Agar No.2 and incubation. The resultant growth was then subject to a Gram stain, as well as a catalase and slide-coagulase tests. The presence of DNAase was confirmed via the addition of hydrochloric acid to the DNAase Agar.

### Rectal swabs for extended spectrum β- lactamase (ESBL) and CPE

Growth on each patient’s clinical specimen’s resultant ESBL and KPC Agar plates, and their respective MacConkey Agar plates, were compared. Further testing was performed for the detection of various types of enzymatic resistance mechanisms, on organisms with a resistant antibiogram profile.

*Stenotrophomonas maltophilia* (*S. maltophilia)* could only be identified using the automated VITEK®2-compact with antibiotic sensitivity testing for co-trimoxazole, chloramphenicol, levofloxacin and colistin performed using Liofilchem® minimum inhibitory concentration (MIC) Test strips as per local protocol. The MICs were interpreted using EUCAST breakpoints for co-trimoxazole and Clinical and Laboratory Standards Institute (CLSI) breakpoints for the remaining 3 antibiotics tested.

### ESBL/AmpC–further testing

ESBL and AmpC screen kit were used in the case of *Escherichia coli* and *Klebsiella pneumoniae*, and cefepime/cefepime and clavulanic acid and cefotetan/cefotetan and cloxacillin MIC Test strips for other members of the Enterobacteriaceae for confirmation of the presence of ESBL/AmpC.

### CPE- further testing

The presence of carbapenemase production was elicited using the Modified Hodge Test (MHT) and confirmation of ertapenem MIC using Liofilchem®MIC test strips. If positive KPC/MBL and OXA-48 Confirm Kit were utilised as a means of identification of enzyme-type.

### Non-fermenters- further analysis

Cases of *Pseudomonas aeroginosa* and *Acinetobacter baumannii* cultivated with raised MICs to carbapenems, were analysed for metallo-β-lactamase (MBL) production.

All of the culture results, as well as the data gathered, were inputted on a central database (WHONET) and subject to an analytical process.

## Results

### MRSA

**Fig. 1:**
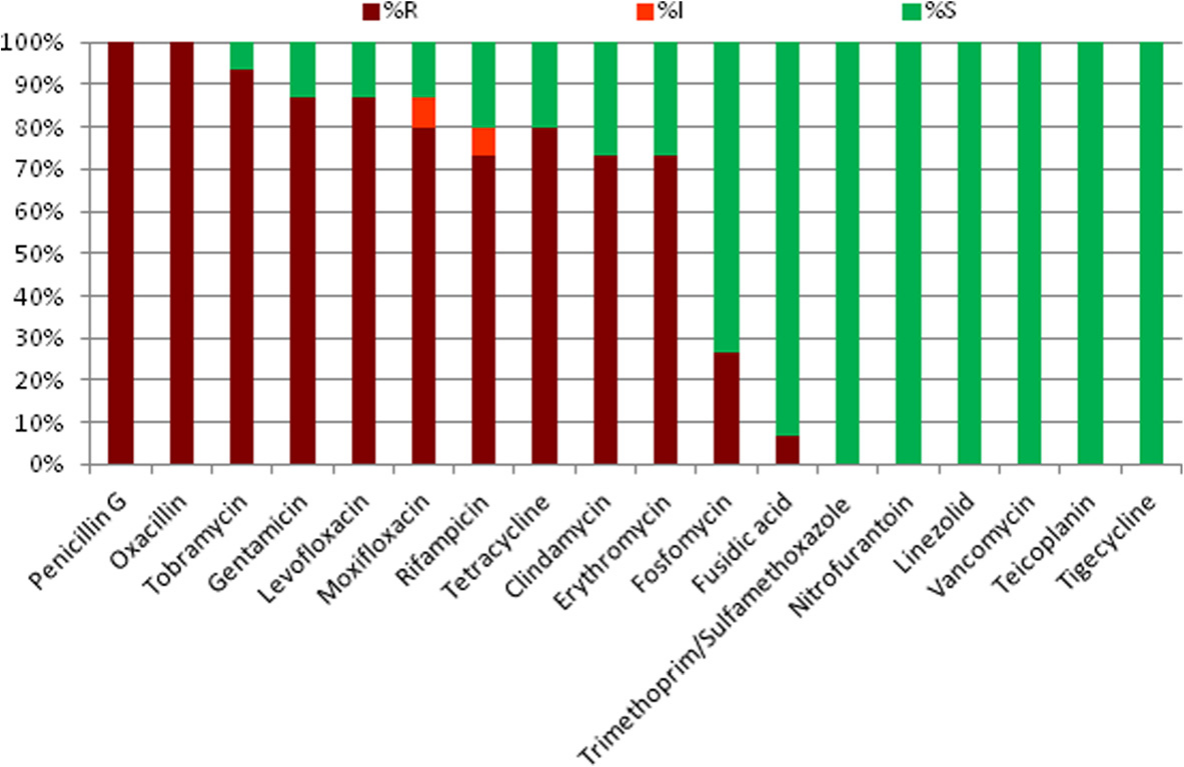
MRSA antibiogram (S = susceptible; I = intermediate; r = resistant)

A total of 106 patients, from 16 wards, were screened for nasal carriage of MRSA, of whom 15 (14.2 % [95 % Confidence Interval (95 CI)] 8.1–22.3 %) were found to be positive for MRSA. In one particular ward 6 out of 9 screened patients were MRSA positive. The resistance profiles for MRSA isolates (*n* = 15) are shown in Table 1. The majority of isolates were resistant to nine or more antibiotic groups tested. Figure 1 shows the percentage susceptibilities for the antibiotics tested in MRSA isolates. Notably, there was a high resistance to the aminoglycosides (gentamicin 86.7 %; tobramycin 93.3 %) and the fluoroquinolones (moxifloxacin 80 % {+6.7 % intermediate}; levofloxacin 86.7 %); none of the MRSA isolates were resistant to glycopeptides, nitrofurantoin and the relatively newer agents, tigecycline and linezolid.

**Table 1:** Resistance profile of MRSA isolates

Resistance profile												*N*° of isolates	% Isolates	*N*° of patients	% Patients
PEN	OXA	–	TOB	–	–	–	–	–	–	–	–	1	6.7	1	6.7
PEN	OXA	–	–	LVX	MFX	–	–	–	–	–	–	1	6.7	1	6.7
PEN	OXA	GEN	TOB	–	–	–	–	–	–	–	–	1	6.7	1	6.7
PEN	OXA	GEN	TOB	LVX	MFX	–	–	TCY	FUS	–	RIF	1	6.7	1	6.7
PEN	OXA	GEN	TOB	LVX	MFX	ERY	CLI	TCY	–	–	RIF	7	46.7	7	46.7
PEN	OXA	GEN	TOB	LVX	MFX	ERY	CLI	TCY	–	FOS	RIF	4	26.7	4	26.7

*Abbreviations*: *PEN* penicillin G, *OXA* oxacillin, *GEN* gentamicin, *TOB* tobramycin, *LVX* levofloxacin, *MFX* moxifloxacin, *ERY* erythromycin, *CLI* clindamycin, *TCY* tetracycline, *FUS* fusidic acid, *FOS* fosfomycin, *RIF* rifampicin

### Enterobacteriaceae

The same wards that had a high rate of MRSA carriage also tended to have a high rate of MDR Enterobacteriaceae (4 of 9 patients). The more commonly identified enzymes were ESBLs, which was detected in 43 of the Enterobacteriaceae isolates.

A total of 104 patients were screened for rectal carriage of multidrug-resistant Gram-negative organisms. Some of these samples yielded more than one such organism. A total of 50 Enterobacteriaceae were isolated from 33 patients [31.7 % (95 CI: 22.9–41.6 %)]. There was heterogeneity in resistance mechanisms identified (Table 2). The overall prevalence of ESBL production was 41.3 % (95 CI: 31.8–51.4 %) i.e., (*n* = 43) from 104 patients. The two more commonly isolated Enterobacteriaceae were *E. coli* (*n* = 28), (24 ESBL positive; 1 AmpC positive and 3 with an unidentified mechanism of resistance) from 22 patients and *Klebsiella pneumoniae* 13 isolates (all ESBL positive; 1 also AmpC and MBL positive) from 10 patients. The *E. Coli* isolates were all resistant to ampicillin but all susceptible to ertapenem, imipenem, meropenem and fosfomycin. The *K. pneumoniae* isolates were also resistant to ampicillin and ceftazidime. Two *Enterobacter cloacae* isolates were ESBL positive whilst the other 2 were ESBL negative but carbapenemase (KPC) and AmpC positive. One *E. coli* was only AmpC positive and one *K. pneumoniae* was both an ESBL, AmpC and carbapenemase (MBL) producer. The other Enterobacteriaceae isolates (*n* = 9) were: *E. cloacae* [*n* = 4 (3 patients)]; *Citrobacter freundii* [*n* = 3]; *Morganella morganii* [*n* = 1]; and *Raoultella planticola* [*n* = 1].

**Table 2:** Resistance profile of Enterobacteriaceae

Organism	No enzyme detected	ESBL (Only)	ESBL (CTXM)	Carbapenemase (KPC) + AmpC	ESBL, AmpC, carbapenemase (MBL)	ESBL, AmpC	AmpC (Only)	Number of organism (Patients)
*Citrobacter* *ferundii*	1	2	–	–	–	–	–	3 (3)
*Enterobacter* *cloacae*	–	2	2	–	–	–	–	4 (3)
*Escherichia* *coli*	3	23	1	–	–	–	1	28 (22)
*Raoultella* *planticola*	–	1	–	–	–	–	–	1 (1)
*Klebsiella* *pneumoniae*	–	12	–	–	1	–	–	13 (10)
*Morganella* *morganii*	–	–	–	–	–	1	–	1 (1)

*Abbreviations*: *ESBL* extended spectrum beta-lactamase, *MBL* metallo beta-lactamase,*KPC Klebsiella pneumoniae* carbapenemase

**Fig. 2:**
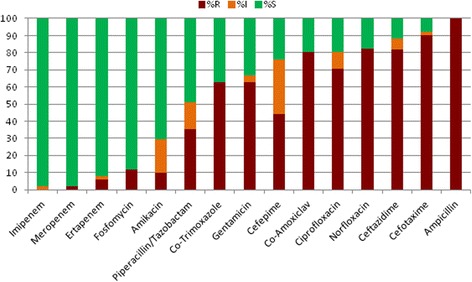
Enterobacteriaceae antibiogram (S = susceptible; I = intermediate; r = resistant)

Figure 2 shows the percentage susceptibilities for all Enterobacteriaceae for the antibiotics tested. This figure clearly indicates a high susceptibility to carbapenems (≥98 %) and an acceptable susceptibility to fosfomycin (90 %) and amikacin (70 %). However, a high level of resistance to all other agents including: the third generation cephalosporins (≤12 % susceptible); gentamicin (34 %) and fluoroquinolones (≤18 %) was observed.

### Non-fermenting gram-negative bacilli

A total of 22 MDR Non-Fermenting Gram-negative bacilli were isolated. Unlike with MRSA and Enterobacteriaceae a different ward had the higher prevalence with *S. maltophilia* (*n* = 2), *Pseudomonas aeruginosa* (*n* = 2) and *Acinetobacter baumannii* (*n* = 1). *S. maltophilia* were only tested for chloramphenicol, colistin, co-trimoxazole and levofloxacin. Of the five *S. maltophilia*, isolated from the 104 patients, 1 tested resistant to chloramphenicol and another to colistin.

The eight *P. aeruginosa* isolates showed a high resistance to ciprofloxacin (75 %) and the aminoglycosides (tobramycin, gentamicin 75 %; amikacin 37.5 %) amongst the isolated strains. Furthermore, all isolates were resistant to meropenem, aztreonam, ticarcillin and ticarcillin-clavulanate. A metallo-β-lactamase (MBL) carbapenemase, was detected in 6 of the 8 *P. aeruginosa* isolates. One of the *P. aeruginosa* isolates was resistant to all antibiotics tested except aztreonam, which was intermediate.

**Fig. 3:**
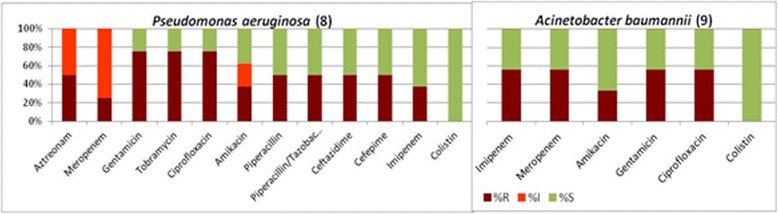
Antibiograms for Pseudomonas aeruginosa and Acinetobacter baumannii. (S = susceptible; I = intermediate; r = resistant)

Figure 3 shows the antibiograms for *P. aeruginosa* and *A. baumannii*. In 5 of the 9 *A. baumannii* isolates an MBL was detected. All *A. baumannii* isolates were susceptible to colistin, 3 were resistant to the carbapenems, ciprofloxacin and the glycopeptides and 2 were resistant to the carbapenems, ciprofloxacin and gentamicin (i.e., susceptible to amikacin).

## Discussion

To our knowledge this is the first prevalence survey on MDRO carriage in patients admitted at QSUT. QSUT is the only reference centre in Albania for acute and critical patients. This survey enabled us to estimate, for the first time, the extent of nasal MRSA and rectal multidrug-resistant Gram-negative organisms (MRGN) carriage in this Albanian referral centre. In a previous study of HAIs carried out at QSUT, Gram-negative bacteria were the predominant pathogens, although *S. aureus* (including MRSA) was the single most frequently isolated pathogen [9].

Our findings on MRSA carriage compare well with the findings from 26 Serbian hospitals, which had a prevalence of 11.8 % in screened patients [10]. Other Balkan reports range from 5.2 % in Croatia [11] and 20 % in Greece [12].

In addition, seven of the 15 MRSA strains showed resistance to aminoglycosides (gentamicin and tobramycin), fluoroquinolones (levofloxacin and moxifloxacin), erythromycin and clindamycin. This could possibly be related to a high use of fluoroquinolones and “macrolides and lincosamides“in Albania both in hospitals and possibly also in the community. Indeed it has been reported that the use of both has been increasing in the community [13].

Our data suggest that MRGN carriage is a greater problem than MRSA. However, there were just three Enterobacteriaceae isolate (1 *K. pneumonia*e and 2 *E. cloacae*) that were resistant to ertapenem implying that CPE does not appear to be as yet a major issue in Albania. Carbapenems are not highly utilised in QSUT, thereby, these results are not surprising considering that there is a link between carbapenem use and resistance in Enterobacteriaceae [14, 15]. These results would support the recently published EUSCAPE report, suggesting that Albania does not seem to be a high prevalence region for New Delhi metallo-β-lactamase (NDM-1) [7, 16]. Our results highlight the need for a sustained surveillance programme of antimicrobial resistance with baseline data on consumption, escalation of infection prevention initiatives and instituting an antimicrobial stewardship programme at QSUT. Measures are also needed so as to increase the hospital’s laboratory functionality. All the latter including an increase in infection control initiatives even of the more basic nature such as, hand hygiene are extremely challenging since the necessary facilities are not universally accessible. Making alcohol based hand-rub available is a fundamental infection prevention recommendation. Moreover, lack of personal protective equipment (PPE) increases the risk of transmission of MDROs within this healthcare setting.

This study had a number of limitations; being a PPS the resistance profiles might have been over represented as the patients with higher risk of carriage were selected. Furthermore, patient participation was non-randomly selected; despite the fact that voluntary participation could be a limitation, only 2 patients refused rectal screening. In addition, we were not in a position to examine whether there was an outbreak or any cross-transmission of MRSA and MDRGN in one of the wards in which a higher number of patients turned out as positive for both MRSA and MDRGN. We had no funds to carry out any typing of the strains isolated.

## Conclusions

In the context of the international problem of ever growing numbers of MDROs, this study has given us a glimpse of the current situation, with respect to MDRO carriage, in one of the major hospital centres in Albania. The study’s findings should serve as guidance and help to target formulation of various initiatives in terms of infection control and antimicrobial stewardship, to help limit the development and spread of MDROs.

## Abbreviations

AMR, Antimicrobial resistance; CLSI, Clinical and Laboratory Standards Institute; CPE, Carbapenemase-producing *Enterobacteriaceae*; ESBL, Extended Spectrum Beta-Lactamase; EUCAST, European Committee on Antimicrobial Susceptibility Testing; HAI, Healthcare Associated Infections; MBL, metallo-β-lactamase; MDR, Multiple-Drug-Resistant; MDH, Mater Dei Hospital; MIC, minimum inhibitory concentration; MHT, Modified Hodge Test; MRSA, methicillin-resistant Staphylococcus aureus; MDRO, multidrug-resistant organisms; MRGN, multidrug-resistant Gram-negative; New Delhi metallo-β-lactamase (NDM-1) PPS, point prevalence survey; QSUT, The University Hospital Centre ‘Mother Teresa’ of Tirana; *S. maltophilia*, *Stenotrophomonas maltophilia*
